# On the existence of a perennial river in the Harappan heartland

**DOI:** 10.1038/s41598-019-53489-4

**Published:** 2019-11-20

**Authors:** Anirban Chatterjee, Jyotiranjan S. Ray, Anil D. Shukla, Kanchan Pande

**Affiliations:** 1 Physical Research Laboratory, Navrangpura, Ahmedabad 380 009 India; 2 Department of Earth Sciences, Indian Institute of Technology Bombay, Powai, Mumbai 400 076 India; 3 Present Address: Department of Geology, Presidency University, Kolkata, 700 073 India

**Keywords:** Palaeoclimate, Geochemistry, Geomorphology, Sedimentology

## Abstract

The legendary river Saraswati of Indian mythology has often been hypothesized to be an ancient perennial channel of the seasonal river Ghaggar that flowed through the heartland of the Bronze Age Harappan civilization in north-western India. Despite the discovery of abundant settlements along a major paleo-channel of the Ghaggar, many believed that the Harappans depended solely on monsoonal rains, because no proof existed for the river’s uninterrupted flow during the zenith of the civilization. Here, we present unequivocal evidence for the Ghaggar’s perennial past by studying temporal changes of sediment provenance along a 300 km stretch of the river basin. This is achieved using ^40^Ar/^39^Ar ages of detrital muscovite and Sr-Nd isotopic ratios of siliciclastic sediment in fluvial sequences, dated by radiocarbon and luminescence methods. We establish that during 80-20 ka and 9-4.5 ka the river was perennial and was receiving sediments from the Higher and Lesser Himalayas. The latter phase can be attributed to the reactivation of the river by the distributaries of the Sutlej. This revived perennial condition of the Ghaggar, which can be correlated with the Saraswati, likely facilitated development of the early Harappan settlements along its banks. The timing of the eventual decline of the river, which led to the collapse of the civilization, approximately coincides with the commencement of the Meghalayan Stage.

## Introduction

The occurrence of a large number of Harappan settlements along the banks of the Ghaggar-Hakra stream, which had remained a seasonal river for most part of its historical existence, has baffled archeologists since the 1950s. Since availability of water is the key to development of a stable urban civilization, many believe that the Ghaggar-Hakra river had a strong fluvial presence during the Harappan times (5.6-3.9 BCE)^1–4^. Interestingly, the Ghaggar-Hakra river-system also shares identical geographic position with the legendary glacier-fed river Saraswati mentioned in some of the ancient Indian scriptures (Rig-Veda, Mahabharata) and often been correlated with the latter^1,2,4–7^. Whereas changing climate is considered to be the primary cause for the deurbanization of the Harappans^8–11^, competing hypotheses consider disorganization of the Ghaggar-Hakra channels to be responsible for the end of the civilization^3,12–14^. Recent geochronological, geochemical and geophysical studies hypothesize that the rivers Yamuna, Sutlej and Beas were probably flowing into the palaeo-Ghaggar and that their migrations disconnected the river from its perennial glacial sources^8,15–17^. It has been proposed that owing to river avulsions, the Yamuna moved away during 49-10 ka and the connection to the Sutlej was lost during the Early-Holocene (~9 ka)^15,16,18^. These events suggest that the major paleo-channels of the northwestern India, identified through remote sensing^5,6^, dried up long before the Harappans settled in this region. Some of these studies even suggest that the lack of perennial flow of the river stabilized the landscape for the civilization to flourish^11,16^.

**Figure 1:**
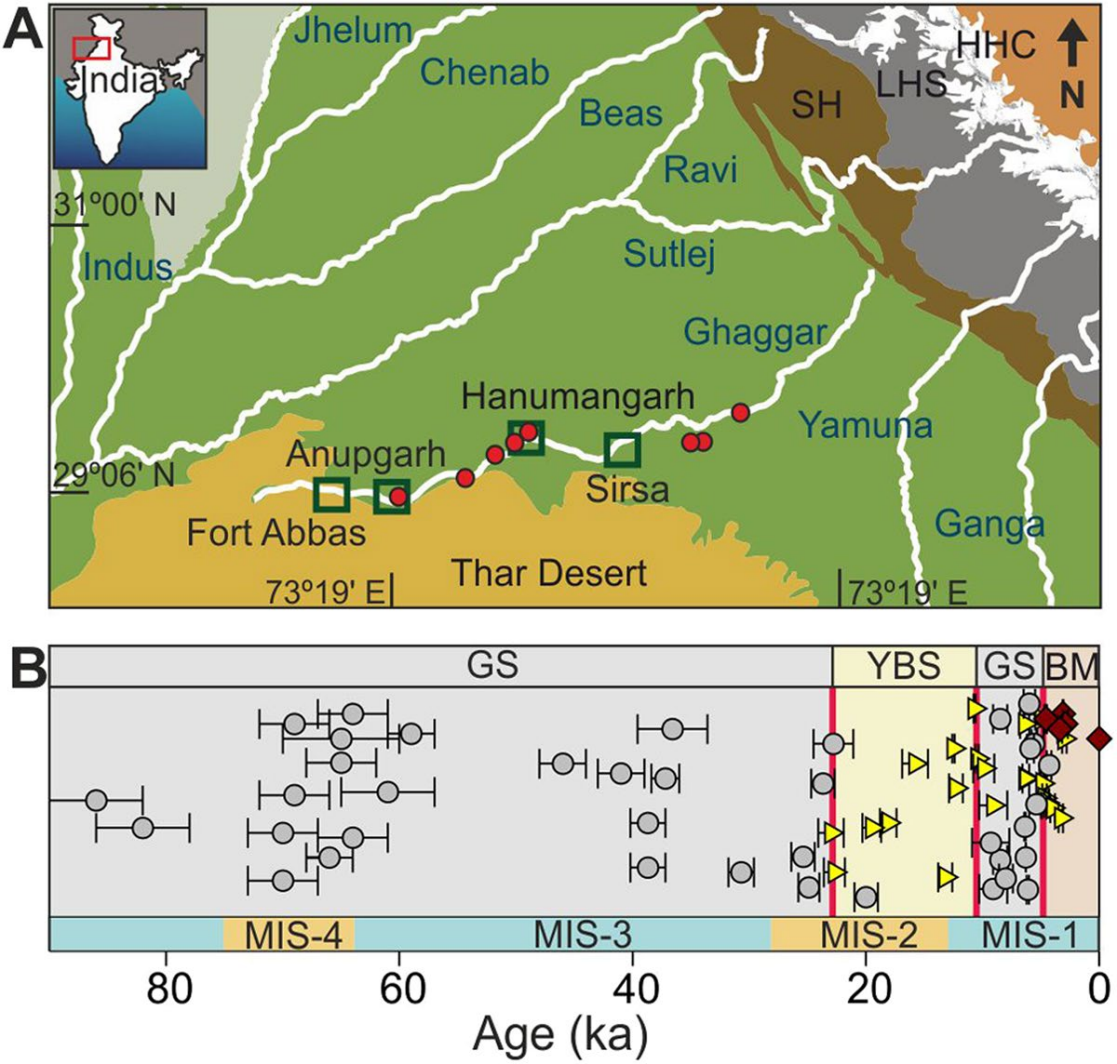
Study area and subsurface stratigraphy along the river Ghaggar. (**A**) Schematic geographical map of north-western India and eastern Pakistan showing drainage basins of the Himalayan rivers. Red dots are studied sections and open squares are major cities along the river Ghaggar. HHC: Higher Himalayan Crystalline, LHS: Lesser Himalayan Series, SH: Siwalik Himalaya. (**B**) Composite stratigraphy of the Ghaggar alluvium based on field and age data from this and earlier studies. GS: Grey Sand (circles); YBS: Yellow Brown Sand (triangles); BM: Brown Mud (diamonds); MIS: Marine Isotopic Stages.

Whereas perennial water availability may not have been the major controlling factor for the evolution of the Harappan settlements along the banks of the Ghaggar^18^, the proposal that the river was ephemeral prior to and during the Harappan period^18^ raises more questions than answers. The observation that the Harappans in the Ghaggar valley made little effort to harvest rain-water, unlike their counterparts in the semi-arid Saurashtra and Rann of Kachchh regions^19^, in spite of the weakening of Indian Summer Monsoon (ISM) since ~7 ka^10,20^, raises serious doubt about the conclusion that the Ghaggar had seasonal water supply. Besides, the extraordinary argument that a dried river system was the best place for the Harappans to colonize, cannot explain the fact that two of their largest cities, Mohenjodaro and Harappa, and many other smaller settlements were built along mighty and frequently flooding Indus and Ravi, respectively. Considering that the pre-Harappan settlements proliferated in the alluvial plains of the north-western Indian subcontinent for at least two millennia, followed by seven centuries of urbanization without a break^7,10,19^, it appears that the Harappan settlements in the Ghaggar valley had thrived with only limited water supply from a petty seasonal stream. Although, evidence for localized increase in rainfall for a few centuries has been reported in the region during the onset of the urban locales^20^, it alone could not possibly have sustained the widespread Pre-Harappan settlements. Therefore, the important question that needs to be asked is: what made the early settlers to build their cities along a supposedly dying river instead of the well-watered plains of neighboring perennial Himalayan Rivers. In an attempt to answer this question and resolve issues related to the existence of the river Saraswati, we investigated the nature of the Ghaggar-Hakra river system during the Holocene by studying the changing provenance of its alluvium through time, at strategic locations (Fig. 1A). Details of sampling, analytical techniques and data used in this study are provided in the supplementary material.

## Results and Discussion

### Stratigraphy of the Ghaggar alluvium

We established the subsurface stratigraphy of the Ghaggar alluvium along an ~300 km stretch of the basin based on field and age data from this work and earlier studies (Fig. 1B; Supplementary Fig. S1). Sedimentological analyses along the river valley helped us ascertain the river’s palaeo morpho-dynamics. The alluvium consists of three distinct sedimentary facies; grey micaceous coarse/medium grained sand (GS; grain size >250 μm), yellowish-brown fine-grained sand (YBS; grain size <125 μm) and brown colored mud (BM), deposited from bottom to top. The GS facies is composed of quartz, feldspar, heavy minerals (zircon, amphibole, kyanite, sillimanite, garnet, and pyroxene)^21^ and mica, which gives it a salt and pepper appearance. It contains poorly sorted coarse to medium, angular sand grains and no clay, which suggest their deposition as fluvial channel-fill/bar deposits under strong hydrodynamic conditions. The textural pattern of the GS facies resembles that of typical channel fills in neighboring Himalayan rivers^16,22^. The fining upward YBS facies contains clay, apart from fine sand and is overlain by the clay dominated BM facies.

The sharp changes in grain size, sorting and clay content from the GS facies to the overlying YBS and BM facies are indicative of the transformation of the fluvial system from a high-energy domain to a low-energy one. Similar observations have been made by many earlier studies in the Ghaggar alluvium^16,21,23^. However, what these studies lacked is the chronology of depositions of these three facies, which is presented in this work. We determined the timings of depositions of various facies by AMS C-14 dating of mollusk shells and optically-stimulated luminescence dating of sands (Supplementary Tables S1 and S2). Our results in conjunction with the existing information suggest that the suspended load, the BM facies, dominated the Ghaggar system since ~4.5ka, whereas sediments of the YBS facies got transported by the river during 20 and 3 ka (Fig. 1B)^16,24,25^. The deposition of the GS facies, on the other hand, can be traced back to the Pleistocene (Fig. 1B). Whereas most studies in the Ghaggar alluvium report cessation of deposition of the GS facies prior to the Holocene, a few report much younger occurrence of the GS facies, in the upper Haryana plains^21,24^. The lack of report of the younger phase of the GS facies in most of the earlier investigations could possibly have been due to sampling bias in a highly meandering fluvial system containing multiple buried channels. With these new data it can be clearly established that the GS facies occur in two time domains, during >80-20 ka and 9.0-4.5 ka with a break of about eleven millennia, along a long stretch of the Ghaggar palaeo-channel (Fig. 1B). The pre-Holocene deposits of the GS facies is generally accepted to be the relicts of a mega Himalayan paleo-river system^15,16^, however, the nature of the younger phase of the GS facies has not yet been explored. In this work we make an attempt to establish the origin of these Holocene sediments of the Ghaggar.

### Provenance of the Ghaggar sediments

**Figure 2:**
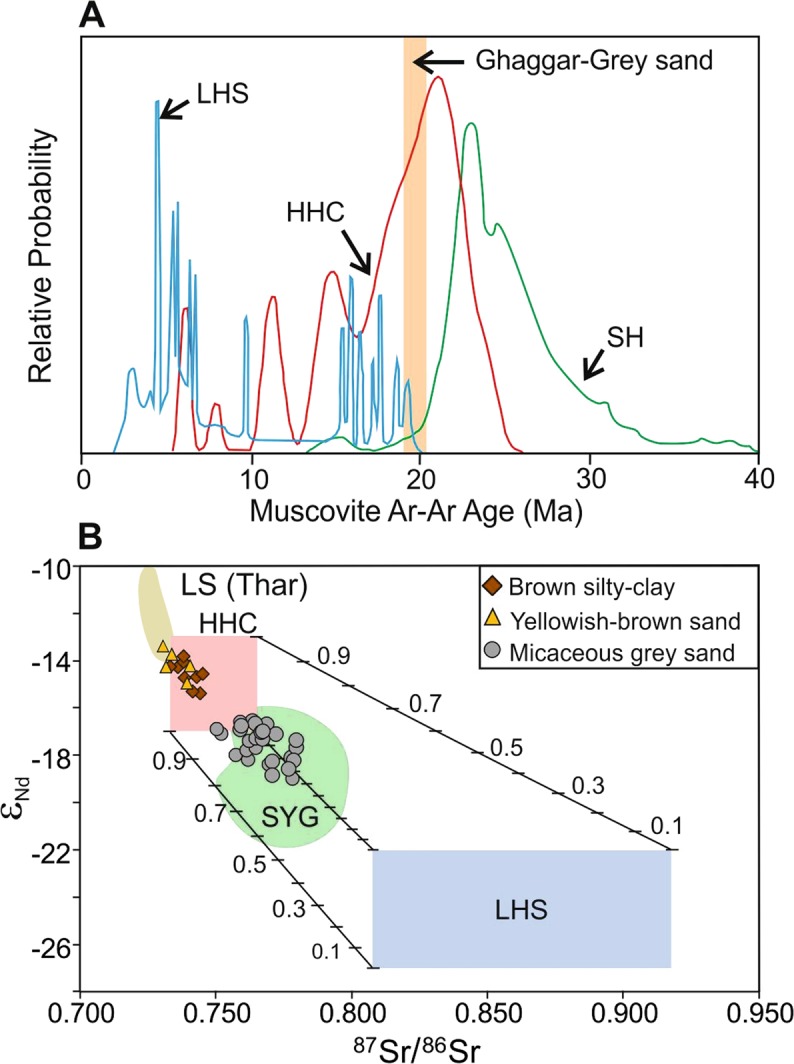
Source fingerprinting with ^40^Ar/^39^Ar muscovite ages and Sr-Nd isotopic ratios of sediments. (**A**) Probability density plot of ^40^Ar/^39^Ar ages of muscovites from different Himalayan litho-tectonic units^40^ compared with our data from detrital muscovites from the Ghaggar alluvium - the orange band (18.6–20.1 Ma). (**B**) ε_Nd_ vs. ^87^Sr/^86^Sr plot of sediments from the Ghaggar alluvium compared with binary mixing curves for the Higher Himalayan Crystalline (HHC) and the Lesser Himalayan Series (LHS) end-members^46^. LS: Local Sources in the Thar Desert^27^. SYG: Sutlej-Yamuna-Ganga.

Since the coarse sands of the GS facies were deposited by a powerful channel of the river in the entire stretch of the alluvium (~300 km), it is reasonable to expect that the sediments were transported from a long distance (>600 km), most likely from the Himalayas. To determine the location of the sources, we utilized ^40^Ar/^39^Ar ages of coarse-grained muscovites present in these sands as provenance indicator. We dated three multigrain separates from the two depositional time intervals to capture the dominant age groups and their temporal distribution. Indistinguishable plateau and isochron ages, and intercepts showing atmospheric ^36^Ar/^40^Ar suggest that these muscovites were derived from igneous or metamorphic rocks which had reached argon closure temperature during 20.1-18.6 Ma (Supplementary Fig. S2). Presence of identical age mica clusters in both the sand horizons (Fig. 2A) suggests their derivation from similar sources. This age has a significant overlap with the mode of the ^40^Ar/^39^Ar age distribution for the muscovites in the Higher Himalayan Crystalline rocks (Fig. 2A), which establishes a most probable genetic link. The dominant source lithologies that could have provided the micas are the 20 ± 2 Ma leucogranites and associated migmatites located above the Main Central Thrust, in the glaciated regions of the Higher Himalaya^26^. The distribution of muscovite ^40^Ar/^39^Ar ages of the Lesser Himalayan rocks also shows a mode, albeit minor, at ~20 Ma (Fig. 2A), therefore, we cannot completely rule out the contribution of sediments from these rocks to the GS facies in the Ghaggar palaeo channels. The rocks of the Siwaliks are unlikely sources of the GS facies sediments, because the detrital muscovites in these rocks are much older than that found in the latter (Fig. 2A). In addition, removal of these mineral grains from the Siwalik rocks could only have contributed weathered mica grains or clays to the latter, which does not appear to be the case. An earlier study of ^40^Ar/^39^Ar ages of detrital mica from the Ghaggar alluvium^16^ had yielded similar results as ours.

**Figure 3:**
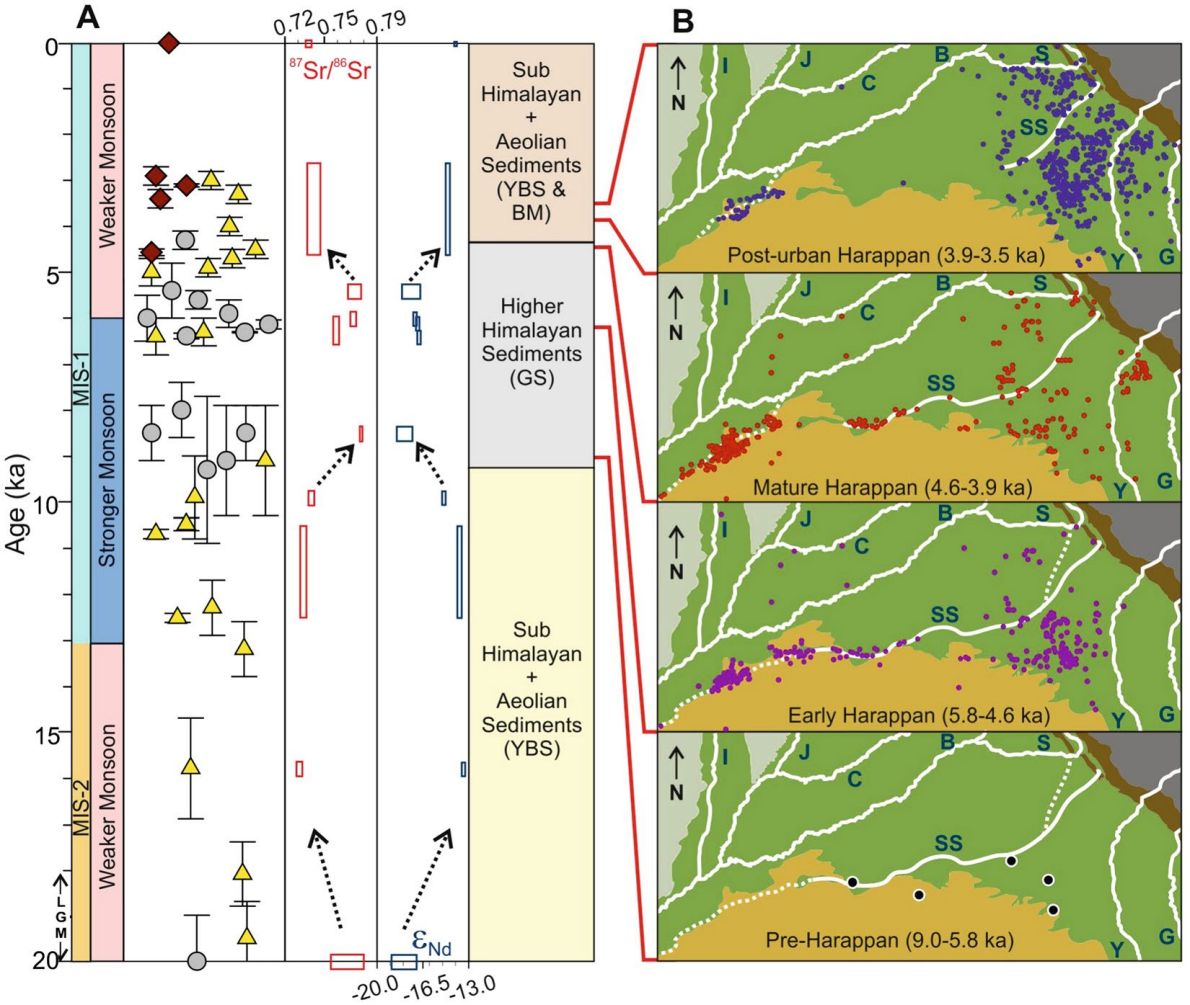
Evolution of the Ghaggar from changes in sediment provenance and the Harappan settlement dynamics. (**A**) Stratigraphic changes in sediment Sr-Nd isotopic compositions in the Ghaggar alluvium during last 20 ka. Symbols and abbreviations are as in Fig. 1. (**B**) Evolution of the Harappan civilization in north-western India and eastern Pakistan as inferred from the settlement dynamics through ages (9.0-3.5 ka)^7,10,47,48^. Modern and inferred former courses (dashed lines) of the major Himalayan rivers are also shown. I: Indus; J: Jhelum; C: Chenab; B: Beas; S: Sutlej; SS: Saraswati (Ghaggar); Y: Yamuna; G: Ganga.

To further constrain the provenance of the Ghaggar alluvium, we used Sr and Nd isotopic ratios of sediments for source fingerprinting (Supplementary Table S3). We found that the sediments in the GS facies horizons, irrespective of their depositional ages, have high ^87^Sr/^86^Sr (>0.75) and low ε_Nd_ (<−17). These compositions overlap with that of sediments in the glacier-fed rivers like the Sutlej, Yamuna, and Ganga, which hint at their derivation from similar sources (Fig. 2B). A binary mixing model using possible Himalayan sources (Fig. 1A) suggests that the GS facies sediments had their sources in the Higher and Lesser-Himalayas (Fig. 2B). In contrast, the sediments of the BM and the YBS facies, having much different isotopic compositions (Fig. 2B), were likely derived from sources made up of already recycled sediments such as the Siwaliks, older alluvium and dunes of the Thar Desert (Fig. 1A)^15,27^. The dominance of smectite over illite in the clay mineral fraction of the YBS facies also supports a local origin for these sediments^27^, since smectite, a secondary clay, was believed to have been produced within the Ghaggar alluvium as a result of the second stage of chemical weathering process^27^. Isotopic data considered in conjunction with the ^40^Ar/^39^Ar ages of the detrital muscovite indicate that the GS facies sediments were directly deposited by a river which had its catchment in the Higher and Lesser Himalaya. An earlier provenance study in the Ghaggar alluvium based on detrital zircon U-Pb geochronology had also traced the sources of the GS facies to the Higher Himalayas^16^. However, the youngest GS facies horizon reported by these authors was older than 12.3 ka^16^, whereas we have encountered and studied much younger horizons of the facies (Fig. 1B; Fig. 3A; Supplementary Fig. S1).

### The evolving river system

A comprehensive reconstruction of the fluvial history of the Ghaggar, based on existing knowledge and results of this study (Fig. 1B; Fig. 3A), reveals that the river was perennial during the Marine Isotope Stages (MIS) 3 and 4 (80-20 ka), when it transported coarse-grained micaceous sand (GS facies) from the glaciated regions of the Higher-Himalaya onto the plains of north-western India. This fluvial phase ended during the peak aridity of the Last Glacial Maximum (LGM)^25^, which also had adversely affected the discharge in other major western-Himalayan rivers^8^. Subsequently, during 20-9 ka, the sediment load in the river got overwhelmed with material derived from the Siwaliks/local sources (YBS facies), which is clearly reflected in the shifts in ^87^Sr/^86^Sr and ε_Nd_ of the Ghaggar alluvium (Fig. 3A). With the intensification of the ISM^10^ and melting of the Himalayan glaciers during the MIS-1^28^ the GS facies made a reappearance at ~9 ka and continued its dominance until ~4.5 ka (Fig. 3A). Although, the Ghaggar received sediments from the Higher Himalaya during the river’s rejuvenated phase, there exists no evidence to suggest that any of its modern tributaries originated from glaciers. In this scenario, the possible pathways for delivery of such sediments into the Ghaggar could have been through the neighboring Sutlej and Yamuna, which currently flow through the Higher and Lesser Himalayan rocks. However, since it is believed that Yamuna had abandoned the Ghaggar channel during 49-10 ka^15^, the only remaining pathway for the Higher Himalayan sediments into the Ghaggar during the Holocene could have been the Sutlej. As a matter of fact, muscovites in the present-day Sutlej sand, which is derived from sources in both the Higher and Lesser Himalayas, have a major ^40^Ar/^39^Ar age mode at 16 ± 3 Ma^16^. Supporting evidence for such a hypothesis comes from the similarity of ^87^Sr/^86^Sr of *in-situ* mollusk shells from the grey-sand bodies (0.7184–0.7190; this work) with that of the water of the Sutlej (0.7166–0.7218)^29,30^. The U-Pb ages of detrital zircons from the Ghaggar alluvium also suggest that the majority of zircon populations in the younger deposits were predominantly originated from the Sutlej^15,16^. The Sutlej is known to have had substantial increase in its water and sediment flux in upper reaches during the Holocene^31^, which could have been delivered to the Ghaggar making the latter a perennial stream for the next few thousand years. At ~4.5 ka, ^87^Sr/^86^Sr and ε_Nd_ of the alluvium revert to the pre-perennial values suggesting the end of supply of the Higher Himalayan sediments due to a break in the Sutlej-Ghaggar connection, which turned the Ghaggar into an ephemeral system. This final disruption of the river flow is also reflected in the sediment composition of its purported delta towards further south in the Great Rann of Kachchh^32^.

### Dynamics of the Harappan settlements

A thorough scrutiny of the settlement dynamics of the Harappan Civilization reveals that the timing of the rejuvenated perennial phase of the Ghaggar (9-4.5 ka) coincides with that of the flourishing of the Pre-Harappan and Early Harappan cultures along its banks (Fig. 3B). Towards the end of the Mature Harappan phase (4.6-3.9 ka), there is a clear evidence of human migrations to the lower and upper reaches of the river, leaving the middle part sparsely populated (Fig. 3B), which could be attributed to the disorganization of the river as established in this work. The lower reaches of the river, in the Hakra sector, had possibly remained perennial, through a connection from the Sutlej, supporting mature and post-urban Harappan settlements (Fig. 3B). Our study brings to light the fact that the Harappans built their early settlements along a stronger phase of the river Ghaggar, during ~9 to 4.5 ka, which would later be known as the Saraswati. However, by the time the civilization matured, the river had already lost its glacial connection. These inferences confirm the observation of an earlier study, based exclusively on changes in the settlement patterns, that the Ghaggar first broke up at ~4.6 ka^12^. Interestingly, the timing of the ultimate disruption to the perennial phase of the Ghaggar roughly coincides with the beginning of the Meghalayan Stage (~4.2 ka)^33^. The urban Harappans abandoned their settlements in the Ghaggar valley within next few centuries and the civilisation declined by 3.9 ka. Although, the decline of the civilization in the Ghaggar-Saraswati valley postdates the exceptional changes to the flow of the river, a stronger perennial phase appears to have helped the early societies to sow the seeds of the earliest known civilization of the Indian subcontinent.

### Methodology

In the arid to semi-arid climate of western India, preservation of organic carbon within the sandy sediments is rare. Therefore, for radiocarbon dating to constrain depositional ages we focussed on collection of mollusc shells buried *in situ* along with the sediments. Shells were cleaned with H_2_O_2_ to remove organic matter. Subsequently, they were dipped in 0.1 N HCl for ~5 seconds and washed thoroughly with deionized water to remove (any) altered outermost layers. Sample powders for dating were micro drilled from the umbo regions, which are generally considered resistant to alteration. AMS C-14 dating was done at the Centro Nacional de Aceleradores, Sevilla, Spain^34^, and the ages were calibrated using INTCAL 13^35^. The typical reproducibility (1σ) of our radiocarbon ages is <1% (Supplementary Table S1).

Optically stimulated luminescence (OSL) of quartz grains in a sediment sample is proportional to its burial age. This dating method works with the premise that the quartz grains have negligible inherited OSL signal prior to deposition and that they subsequently acquire it from ambient radiation. The zeroing of OSL signal in these grains happens upon exposure to daylight prior to deposition, while on transport, or during any recycling/re-exposure event. For dating purpose, total OSL of quartz grains, separated from a sample in dark-room conditions, is measured and converted to units of radiation dose or total paleo-dose, which is then divided by the dose rate to derive the age since last exposure. The dose rate is determined from measured concentrations of U, Th and K and estimated contributions from cosmic rays^36^ in the immediate burial environment of the sample. Samples for of this study, collected from freshly cleaned up stratigraphic sections using specially designed aluminium pipes, were dated using the Single Aliquot Regeneration (SAR) protocol for quartz grains^37^. Concentrations of U, Th and K were measured on a High purity Germanium detector (HPGe). The OSL dating was done at the Physical Research Laboratory, India. The typical reproducibility (1σ) of our OSL ages is <10% (Supplementary Table S2).

The closure temperature of the ^40^Ar/^39^Ar isotope system for muscovites is ~350 °C^38^, which enables it to capture the timings of the latest tectonothermal events experienced by the parent rocks. Since the litho-tectonic units of the Himalaya had exhumed diachronously, they contain various age populations of muscovites representing each of the exhumation event^39^. Therefore, the ^40^Ar/^39^Ar ages of detrital muscovites, derived from these rocks, serve as powerful source indicators^40^. To determine ^40^Ar/^39^Ar ages of detrital muscovites in the Ghaggar alluvium we handpicked ~200 mg of grains, with grain size >500 μm, from grey sand horizons from three distinct stratigraphic horizons with depositional ages of 37, >16 and 6.3 ka. Smaller mica grains were avoided because they are more easily affected by secondary alteration as compared to larger grains^41^. Moreover, determination of ^40^Ar/^39^Ar ages of smaller grains is difficult and can become erroneous because of Argon loss due to recoil during irradiation^42^. Samples were packed in aluminium capsules and irradiated in a research reactor by fast neutrons. The Minnesota hornblende reference material (MMhb-1), of age 523.1 ± 2.6 Ma^43^, was used as the flux monitor and high-purity CaF_2_ and K_2_SO_4_ salts for interference corrections arising from the production of Ar from Ca and K isotopes. Argon was extracted by incremental heating between 450 °C and 1400 °C at steps of 50 °C and isotopic ratios were measured in a Thermo Fisher ARGUS-VI multi-collector mass spectrometer at the Indian Institute of Technology Bombay. Plateau and isochron ages were calculated and plotted using the software ISOPLOT 2.49^44^.

Rocks in various sectors of the Himalayan Mountain Belt have distinct groupings in isotopic ratios of Sr and Nd^45^. Therefore, ^87^Sr/^86^Sr and ^143^Nd/^144^Nd ratios of sediments in the Indo-Gangetic plain can be utilized for source fingerprinting or provenance study. Our isotopic analyses were carried out in powdered (to <10 μm) and homogenized samples. Samples were heated to 650 °C for 2 hours to remove organic matter. They were decarbonated using dilute HCl and washed multiple times in deionized water to ensure removal of salts. Thus, further processing for isotopic analyses was done for the silicate fractions only. Samples were dissolved using the standard HF-HNO_3_-HCl dissolution procedure for silicate rocks and separation of Sr and Nd was done by conventional column chemistry^27,41^. ^87^Sr/^86^Sr and ^143^Nd/^144^Nd were measured in static multicollection mode on an Isoprobe-T thermal ionization mass spectrometer (TIMS) and Thermo Neptune Multi-Collector Inductively Coupled Plasma Mass Spectrometer (MC-ICPMS), respectively at the Physical Research Laboratory^27,32^. The average values for NBS987 and JNdi-1 measured on TIMS over a period of 5 years are ^87^Sr/^86^Sr = 0.71023 ± 0.00001 (n = 70) and ^143^Nd/^144^Nd = 0.512104 ± 0.000004 (n = 60; ± 0.1 in ε_Nd_ units) at 2σ level of uncertainty^41^. The average ^143^Nd/^144^Nd of the in-house lab standard, Merck Nd solution, was 0.511705 ± 27 (2σ, n = 56) for the MC-ICPMS measurements. To compare our data with that from literature, all the ^87^Sr/^86^Sr and ^143^Nd/^144^Nd ratios were normalized to 0.71025 for NBS987 and 0.511858 for La Jolla, respectively. All plots and discussion in this work are based on the normalized ratios.

## Supplementary information

Supplementary figures and data

## Data Availability

All data associated with this work are available in the supplementary materials.
