# Supplementary material for: On the existence of a perennial river in the Harappan heartland
Source: Sci Rep. 2019 Nov 20;9:17221. doi: 10.1038/s41598-019-53489-4 (PMC6868222; doi:10.1038/s41598-019-53489-4)
Supplement: Supplementary file 1 — Supplementary figures and data [file 41598_2019_53489_MOESM1_ESM.pdf]

Supplemental Material (figures) for

On the existence of a perennial river in the Harappan heartland

Anirban Chatterjee*, Jyotiranjan S. Ray*, Anil D. Shukla, Kanchan Pande.

*Correspondence to: anirban.geol@presiuniv.ac.in, jsray@prl.res.in

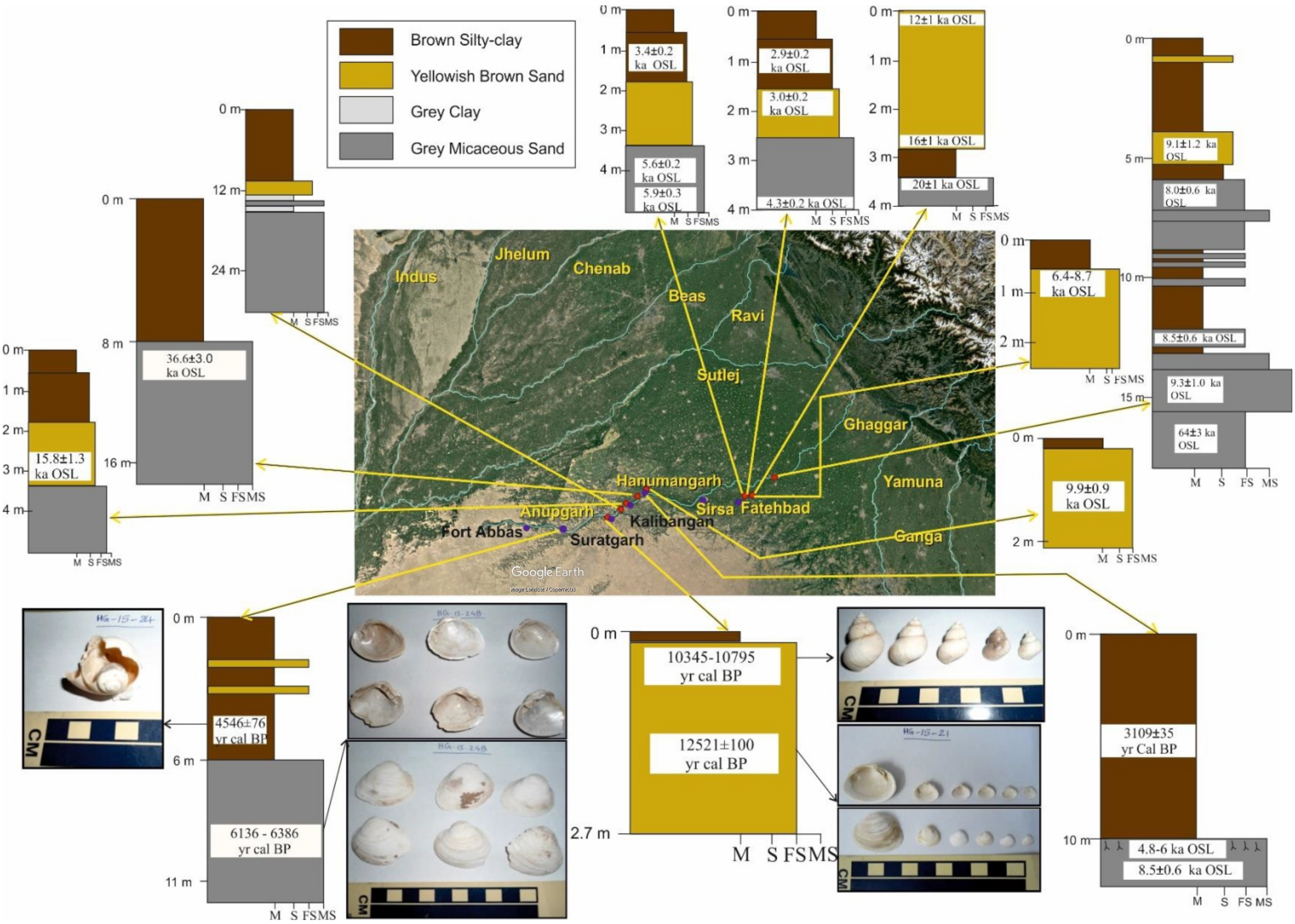

Figure. S1.

A comparison of the subsurface stratigraphy from different localities along the Ghaggar floodplain, constructed using field and age data from the present and earlier studies [1, 2, 3]. Images of different mollusc shells used for AMS C-14 dating are presented adjacent to the horizons from which they were recovered. The Satellite imagery was obtained from the Google Earth and edited using CorelDraw Graphics Suit X6 (http://coreldraw.com)

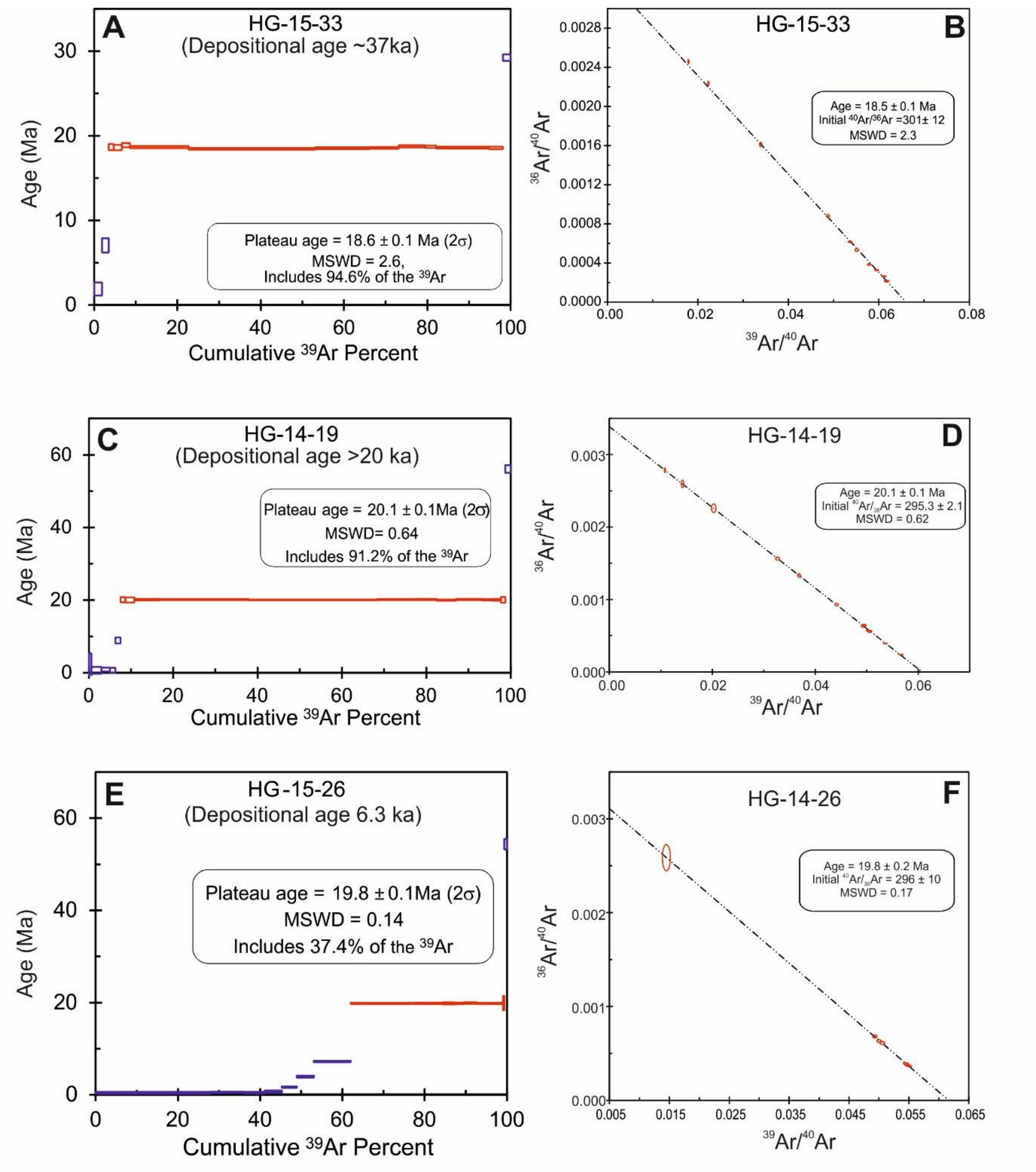

Figure. S2.

^{40}Ar-^{39}Ar plateau and isochron plots for muscovite concentrates from the grey micaceous sand facies in the Ghaggar alluvium.

References

1. Chatterjee, A. & Ray, J. S. Geochemistry of Harappan Potteries from Kalibangan and sediments in the Ghaggar River: Clues for a Dying River. *Geosci. Front.* **9,** 1203–1211 (2018).

2. Singh, A. *et al.* Counter-intuitive influence of Himalayan river morphodynamics on Indus Civilisation urban settlements. *Nat. Commun.* (2017). doi:10.1038/s41467-017-01643-9

3. Saini, H. S., Tandon, S. K., Mujtaba, S. A. I., Pant, N. C. & Khorana, R. K. Reconstruction of buried channel-floodplain system of the northwestern Haryana Plains and their relation to the ' Vedic ' Saraswati. *Curr. Sci.* **97,** 1634–1643 (2009).

Supplemental Material (dataset) for

On the existence of a perennial river in the Harappan heartland

Anirban Chatterjee*, Jyotiranjan S. Ray*, Anil D. Shukla, Kanchan Pande.

*Correspondence to: anirban.geol@presiuniv.ac.in, jsray@prl.res.in

Sample	Sample type	age (y)	±(y)	$\delta^{13}C$ (‰)	Cal BC	±(y)	Cal BP
S-1	Gastropod	4,021	34	-1.3	2,546	76	**4,546**
S-2	Bivalve	5,506	36	-5.1	4,386	62	**6,386**
S-3	Bivalve	5,347	36	-5.7	4,307	14	**6,307**
S-4	Bivalve	5,285	35	-5.1	4,136	98	**6,136**
S-5	Bivalve	10,480	46	-8.2	10,521	100	**12,521**
S-6	Bivalve	9,410	44	-8.6	8,695	100	**10,695**
S-7	Gastropod	9,272	43	-5.6	8,484	139	**10,484**

Table S1.
AMS radiocarbon data for mollusc shells from Ghaggar alluvium

Sample	OD	U (ppm)	Th (ppm)	K (wt%)	De	Dose rate (μGy/a)	Age (ka)
G-7	27.5	4.43±0.07	20.0±0.4	1.43±0.02	20.6±0.7	3.5±0.2	**6.0±0.5**
HG-OSL-6(Chatterjee and Ray, 2018)	13.4	2.65±0.07	12.1±0.3	2.0±0.03	26.4±0.5	3.1±0.2	**8.5±0.6**
HG-OSL-4(Chatterjee and Ray, 2018)	23	3.25±0.09	14.4±0.4	1.73±0.03	49.5±1.3	3.1±0.2	**15.8±1.1**
G15-A(15/5)	29.7	2.20±0.07	10.2±0.4	1.47±0.03	24.3±1.5	2.5±0.2	**9.9±0.9**
G15-C(15/13)	34.6	3.83±0.09	17.5±0.5	1.39±0.03	106±14	3.2±0.2	**36.6±3.0**
G15-D	27	2.65±0.07	12.1±0.3	2.0±0.03	16.8±1.5	3.1±0.2	**5.4±0.6**

OD: Over Dispersion; De: Equivalent Dose.

Table S2.

Parameters used in OSL dating of quartz grains from Ghaggar alluvium

Samples	Brown Silty-clay (<4.5 ka)							
Samples	HG-14-4(Chatterjee and Ray, 2018)	HG-14-8(Chatterjee and Ray, 2018)	HG-14-16(Chatterjee and Ray, 2018)	G4-34	G4-35	G4-38	G4-39	HG-14-20 (Chatterjee and Ray, 2018)
$^{87}Sr/^{86}Sr$	0.743022	0.745287	0.747306	0.744301	0.741571	0.738601	0.736119	0.733185
ε_{Nd}	-14.7	-14.6	-14.8	-15.4	-15.3	-14.7	-14.3	-14.3

Samples	Modern surface mud		Yellowish brown sand (<15 ka)				
Samples	HG-14-18 (Chatterjee and Ray, 2018)	HG-14-18R (Chatterjee and Ray, 2018)	HG-14-21(Chatterjee and Ray, 2018)	HG-14-22(Chatterjee and Ray, 2018)	G4-36	G5-3	G5-19
$^{87}Sr/^{86}Sr$	0.738182	0.738182	0.731733	0.730670	0.740603	0.739478	0.733869
ε_{Nd}	-14.1	-13.8	-14.3	-13.4	-14.2	-14.9	-13.7

Samples	Grey Micaceous sand (9-5 ka)							
Samples	G4-17	G4-19	G4-19R	G4-29	G4-30	G4-31	G4-31R	G4-33
$^{87}Sr/^{86}Sr$	0.768847	0.770905	0.770905	0.777894	0.776909	0.779743	0.779743	0.763566
ε_{Nd}	-16.7	-18.9	-18.3	-18.1	-18.6	-17.7	-17.4	-16.6

Samples	Grey Micaceous sand (9-5 ka)							
Samples	G4-41	G5-10	G5-10R	G5-12	G5-13	G5-24	G5-26	G5-28
$^{87}Sr/^{86}Sr$	0.772213	0.764594	0.764594	0.766860	0.767445	0.759107	0.759457	0.767271
ε_{Nd}	-17.1	-16.6	-16.7	-17.0	-17.0	-16.9	-16.8	-17.3

Samples	Grey Micaceous sand (9-5 ka)	Grey Micaceous sand (>20 ka) (Singh et al., 2016)				
Samples	G5-33	GS11-F17	GS11-F16a	GS11-F16	GS11-F15	GS11-F13
$^{87}Sr/^{86}Sr$	0.778758	0.76962	0.761	0.762	0.77831	0.76472
ε_{Nd}	-18.2	-18.4	-18	-18.2	-19	-17.3

Samples	**Grey Micaceous sand (>20 ka)** (Singh et al., 2016)							
Samples	GS11-F12	GS11-F10	GS11-F9	GS11-F7	GS11-F6	GS11-F5	GS10-F8/F6	
$^{87}Sr/^{86}Sr$	0.75743	0.76137	0.76478	0.76228	0.7521	0.75045	0.75909	
ε_{Nd}	-18	-17.8	-17.7	-17.4	-17.1	-16.9	-16.6	

Table S3. Sr-Nd isotopic compositions of sediments from Ghaggar alluvium

References

Chatterjee, A., and Ray, J.S., 2018, Geochemistry of Harappan potteries from Kalibangan and sediments in the Ghaggar River: Clues for a dying river: Geoscience Frontiers, v. 9, p. 1203–1211, doi: https://doi.org/10.1016/j.gsf.2017.07.006.

Singh, A., Paul, D., Sinha, R., Thomsen, K.J., and Gupta, S., 2016, Geochemistry of buried river sediments from Ghaggar Plains , NW India : Multi-proxy records of variations in provenance , paleoclimate , and paleovegetation patterns in the Late Quaternary: Paleogeography, Paleoclimatology, Paleoecology, v. 449, p. 85–100, doi: http://dx.doi.org/10.1016/j.palaeo.2016.02.012.
